# Supplementary material for: The Integration of a Three-Dimensional Spheroid Cell Culture Operation in a Circulating Tumor Cell (CTC) Isolation and Purification Process: A Preliminary Study of the Clinical Significance and Prognostic Role of the CTCs Isolated from the Blood Samples of Head and Neck Cancer Patients
Source: Cancers (Basel). 2019 Jun 6;11(6):783. doi: 10.3390/cancers11060783 (PMC6627984; doi:10.3390/cancers11060783)
Supplement: Supplementary file 1 [file cancers-11-00783-s001.pdf]

Supplementary Information: The Integration of a Three-Dimensional Spheroid Cell Culture Operation in a Circulating Tumor Cell (CTC) Isolation and Purification Process: A Preliminary Study of the Clinical Significance and Prognostic Role of the CTCs Isolated from the Blood Samples of Head and Neck Cancer Patients

Chia-Jung Liao, Chia-Hsun Hsieh, Feng-Chun Hung, Hung-Ming Wang, Wen-Pin Chou and Min-Hsieh Wu

Table S1. Cancer-related gene expression pattern in HNSCC.

Pt#	Tumor Site	Stage	Disease Progression	E-CTC/M-CTC (Number/mL)	MRP 1	MRP 2	MRP 4	MRP 5	MRP 7	ALDH 1	CD133	NANOG	OCT 4	SOX 2	CK 19	CDH 1	CDH 2	JUP	SNAI 1	TWIST 1	VIM
5	Hypopharynx	IVb		0/0																	
19	Oropharynx	IVc		3/5																	
3	Oropharynx	III	No	0/2.8																	
2	Oropharynx	IVa	No	1/2.1																	
12	Oropharynx	IVa	No	2.2/2.6																	
13	Oropharynx	IVa	No	2.3/4.6																	
4	Hypopharynx	IVa	No	1.1/5.1																	
18	Hypopharynx	IVa	No	0/3.8																	
1	Oral cavity	IVb	No	1.7/12.8																	
8	Oral cavity	IVb	No	6.6/5.5																	
16	Oropharynx	IVb	No	0/3																	
15	Hypopharynx	IVb	No	0/0																	
6	Larynx	IVc	No	24/6																	
11	Larynx	IVc	No	0.6/3																	
20	Hypopharynx	IVc	No	2.4/18																	
14	Oropharynx	IVa	Yes	2/3																	
17	Oropharynx	IVb	Yes	1.2/27.5																	
7	Hypopharynx	IVb	Yes	6.6/3																	
9	Hypopharynx	IVb	Yes	26.5/3.5																	
10	Oropharynx	IVc	Yes	3/17.5																	

Pink box, high expression; green box, low expression.

© 2019 by the authors. Licensee MDPI, Basel, Switzerland. This article is an open access article distributed under the terms and conditions of the Creative Commons Attribution (CC BY) license (http://creativecommons.org/licenses/by/4.0/).
